# Impact of New Chemotherapy Regimens on the Treatment Landscape and Survival of Locally Advanced and Metastatic Pancreatic Cancer Patients

**DOI:** 10.3390/jcm9030648

**Published:** 2020-02-28

**Authors:** Markus Kieler, Matthias Unseld, Daniela Bianconi, Martin Schindl, Gabriela V. Kornek, Werner Scheithauer, Gerald W. Prager

**Affiliations:** 1 Department of Medicine I, Division of Oncology, Comprehensive Cancer Center Vienna, Medical University of Vienna, Währinger Gürtel 18-20, 1090 Vienna, Austria; markus.kieler@meduniwien.ac.at (M.K.); matthias.unseld@meduniwien.ac.at (M.U.); daniela.bianconi@meduniwien.ac.at (D.B.); gabriela.kornek@meduniwien.ac.at (G.V.K.); werner.scheithauer@meduniwien.ac.at (W.S.); 2 Department of Surgery, Division of General Surgery, Pancreatic Cancer Unit, Comprehensive Cancer Center Vienna, Medical University of Vienna, Währinger Gürtel 18-20, 1090 Vienna, Austria; martin.schindl@meduniwien.ac.at

**Keywords:** pancreatic ductal adenocarcinoma, treatment landscape, treatment patterns, treatment sequences, new chemotherapy regimens, nanoliposomal irinotecan, nab-paclitaxel, FOLFIRINOX

## Abstract

Background: New chemotherapy regimens for the treatment of metastatic pancreatic cancer have changed the therapy paradigm. We aimed to assess their impact on the treatment landscape and clinical outcome at our academic institution. Methods: In this single institutional posthoc registry analysis, we assessed characteristics and survival rates from all patients with locally advanced and metastatic pancreatic cancer who started a systemic treatment between 01/2011 and 12/2017. Survival analyses were performed by Kaplan-Meier and Cox proportional hazards model. Results: A total of 301 patients started a systemic treatment in the observation period. In the first line treatment, we observed a shift from the four different main regimens (gemcitabine/nab-paclitaxel, modified FOLFIRINOX, gemcitabine/oxaliplatin +/− erlotinib or gemcitabine alone) to gemcitabine/nab-paclitaxel and modified FOLFIRINOX that add up to more than 80% of administered first line treatments in each of the time cohorts (2011–2013 vs. 2014–2017). The rate for first line modified FOLFIRINOX treatment was balanced between the two groups (19% and 15%). Median overall survival differed significantly between the two time cohorts (8.89 versus 11.9 months, *p* = 0.035). Survival rates for different first to second line treatment sequences (modified FOLFIRINOX to gemcitabine/nab-paclitaxel, gemcitabine/nab-paclitaxel to fluoropyrimidines plus nanoliposomal irinotecan, or gemcitabine/nab-paclitaxel to fluoropyrimidines plus oxaliplatin) were not significantly different and median overall survival ranged from 14.27 to 15.64 months. Conclusion: Our study provides real-world evidence for the effectiveness of the new chemotherapy regimens and underscores the importance of the choice of the front-line regimen when considering different sequencing strategies.

## 1. Introduction

Pancreatic ductal adenocarcinoma (PAC) is projected to become the second leading cause of cancer deaths in the United States, as well as Europe in the next decade. Unlike most other members of the top five cancers with the highest number of cancer deaths, such as colorectal, breast, lung and prostate cancer, its incidence is increasing [1,2]. One reason is that the majority of patients is diagnosed at a metastatic or locally advanced disease stage, due to lack of specific symptoms in early stages and yet no effective screening program [3]. In addition, high relapse rates after resection with curative intent are observed. Thus, the five-year survival rate of pancreatic cancer ranges between~5 to 10% with no raising numbers within the last 20 years [4]. Moreover, aggressive tumor biology and unresponsiveness to most of the currently clinically tested molecularly targeted therapies, as well as immunotherapies, which have revolutionized anti-cancer treatment in many other cancers, contribute to this devastating prognosis [5,6].

Systemic chemotherapy still remains the standard of care for patients with metastatic PAC. In the past, fluorouracil (5-FU) has been the most commonly used chemotherapy regimen until in 1996, gemcitabine (Gem) was approved as it was shown to modestly improve survival and relieve disease-related symptoms [7]. After the introduction of Gem, many different Gem-based combinations have been clinically tested with the majority of trials not demonstrating a meaningful clinical benefit. However, two meta-analyses found that combinations with platins or capecitabine improved the survival of patients with unresectable PAC and good performance status [8,9]. Of note, in 2006, a prospective randomized clinical phase III trial demonstrated that the addition of erlotinib to Gem marginally extended the lives of patients affected from inoperable PAC by only two weeks. So far, this is the only targeted therapy approved for the treatment of advanced PAC [10]. More recently in 2011, the PRODIGE4/ACCORD11 (Partenarait de Recherche en Oncologie Digestive/Actions Concertées dans les Cancers Colorectaux et Digestifs) study evaluated the combination of 5-FU, leucovorin (LV), irinotecan, and oxaliplatin (FOLFIRINOX) versus Gem monotherapy and reported a median overall survival (mOS) of 11.1 months against 6.8 months in the control arm (*p* < 0.001) [11]. Two years later, the MPACT trials also demonstrated superior survival with the combination of Gem plus nab-paclitaxel (Gem/NabP) over Gem alone with an mOS of 8.5 versus 6.7 months (*p* < 0.0001) [12]. The latest approved therapy for the treatment of unresectable PAC is nanoliposomal irinotecan with 5-FU/LV (FP/Nal-IRI) according to the positive results of the NAPOLI-1 trial, which evaluated this regimen in patients that progressed under a Gem based therapy [13]. Another clinically tested second line treatment option after front-line therapy with Gem is fluoropyrimidines in combination with oxaliplatin, although there is conflicting evidence concerning the efficacy of the different regimens [14,15]. With these recent developments in the clinical management of patients with advanced PAC, our aim was to determine the impact of new therapeutic options on the treatment landscape and clinical outcome in a cohort of patients with advanced or metastatic PAC, who have started their treatment at the Comprehensive Cancer Center of the Medical University of Vienna during 2011 and 2017.

## 2. Experimental Section

### 2.1. Study Design

This is a single-center, retrospective, observational registry study, including patients with histologically or cytologically proven non-resectable PAC which was either locally advanced or metastasized and who have started a systemic treatment at the Medical University of Vienna between 01/2011 and 12/2017. The decision for the systemic treatment was always at the discretion of the treating physician. Furthermore, all patients in this study were discussed within multidisciplinary board sessions. If a patient was progressing to frontline chemotherapy and maintained sufficient performance status to undergo a second or later line chemotherapy, potential treatment options, which were available at that time, were discussed with the patient during the informed consent discussion. The decision for second line chemotherapy was not influenced by industry-funded trials, as there were no ongoing second line chemotherapy trials during the whole observation period. The selection of the optimal treatment options was based on the at that time available national and international treatment guidelines. For the comparison of the main study cohorts, the time of first administration of systemic chemotherapy was retrieved. The electronic medical history was queried for patient demographics, performance status, date of diagnosis, date of advanced disease, diagnosis and carbohydrate antigen 19-9 (CA19-9) level at baseline, treatment details and overall survival. ECOG (Eastern Cooperative Oncology Group) performance status was derived, if not stated explicitly, from the medical history, including comorbidities and overall assessment of the treating physician. Recurrent PAC after resection of curative intent was stated as stage IV disease. Date of disease progression on treatment and date of death were recorded. The here presented data analysis received prior approval by the ethical committee of the Medical University of Vienna (EK.No. 1806/2017) and was performed according to Helsinki criteria of good scientific practice.

### 2.2. Statistics

Descriptive statistics were calculated as mean, median or percentages as appropriate. Statistical comparison of categorical variables was calculated with Fisher’s exact test and comparison of metric variables with the unpaired *t* test. Overall survival (OS) from first, second or third treatment line was calculated from the time of the first administration of the respective treatment line to death. OS was depicted by Kaplan Meier plots. For group comparisons, the Log-Rank test (LRt) and the Gehan-Breslow-Wilcoxon test (GBWt) (in the case of non-constant hazard ratios) were used, respectively. Multivariate survival analysis was performed with the Cox proportional hazard model to evaluate the treatment effect with adjustment for stratification factors. In the case of missing variables, the entire data of that individual was deleted from the multivariate analysis. For the covariates ECOG performance status and number of metastatic sites, categories were combined because of the low number of events. A *p*-value of <0.05 was considered as statistically significant, and the term “significant” was used to indicate that the computed *p*-values were below this cut-off value. The assessment of respectively constant and non-constant hazard ratios was based on a graphical approach. No formal sample size calculation has been performed because of the retrospective character of the study. Statistical analysis of Kaplan-Meier plots, Log-Rank test and the Gehan-Breslow-Wilcoxon test was performed with GraphPad Prism Software Prism 7 for Windows, Version 7.03, February 20, 2017 (GraphPad Software, La Jolla, CA, USA). Multivariate survival analysis (Cox proportional hazard model) was performed with R for Windows, Version 3.6.2, as well as RStudio for Windows, Version 1.2.5033.

## 3. Results

### 3.1. Patient Characteristics

Between 2011 and 2017, a total of 301 patients started a systemic treatment, due to recently diagnosed metastatic or unresectable locally advanced PAC. The total cohort was split up into two nearly equally sized cohorts, cohort A (2011–2013, *n* = 132) and cohort B (2014–2017, *n* = 169), according to the time point of initiating systemic chemotherapy for this disease. Table 1 lists patient and tumor characteristics of the two main cohorts.

The median age in both groups was nearly identical (66.3 (interquartile range (IQR)) 57.3–72.2) versus 66.0 years (IQR 58.3–73.3)). In cohort A there were 53 (41%) female and 79 (59%) male patients, while in cohort B there were 90 female (53%) and 79 (47%) male patients. About one fourth in each cohort presented with locally advanced (unresectable) disease (26% and 25%), while the majority had metastatic disease (74% and 75%) at time of initiation with systemic treatment. In cohort A 23 (17%) and in cohort B 32 (19%) patients had a prior surgical resection. The median CA 19-9 level was 505.35 kU/l (68.05–2261.25 kU/l) in cohort A and 686.40 kU/l (88.25–3771.00 kU/l) in cohort B. While a minor proportion in each of the cohorts had CA 19-9 levels within the normal range (15% and 11%), most of the patients had increased CA 19-9 levels at the start of their systemic therapy (77% and 78%). In 8% and 11%, the levels for CA 19-9 were not available. In both cohorts the predominant site of metastatic disease was liver (55% and 50%), followed by lung (11% and 12%), peritoneum (8% and 18%) and other anatomical locations (8% and 4%). Relative numbers for metastatic sites for patients were equivalent. There were 60% with one metastatic site, as well as 11% with two in each cohort, while there were 3% and 4% of patients who had three different organs affected by metastatic spread. Most of the patients presented with good ECOG performance status (90% and 95% with ECOG 0-1) and 6%, as well as 3% had an ECOG performance status of two.

### 3.2. Treatment Landscape

**Figure 1:**
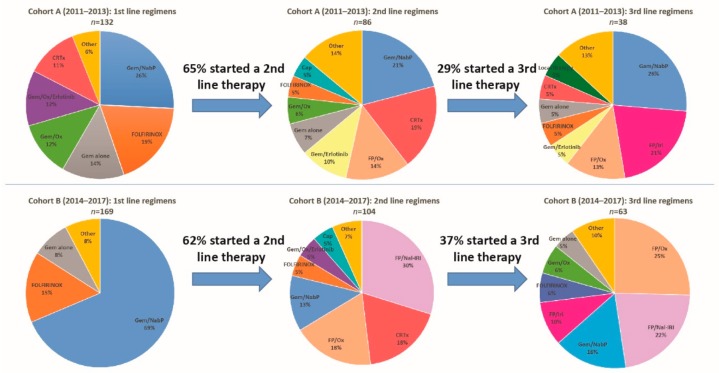
Treatment patterns. Treatment regimens from first to third line of locally advanced or metastatic patients who started a systemic treatment between 2011–2013 and 2014–2017. Abbreviations: Gem/NabP, gemcitabine/nab-paclitaxel; FOLFIRINOX, 5-FU, leucovorin (LV), irinotecan, and oxaliplatin; Gem/Ox, gemcitabine/oxaliplatin; FP/Ox, fluoropyrimidines/oxaliplatin, FP/Iri, fluoropyrimidines/irinotecan; FP/Nal-IRI, fluoropyrimidines/nanoliposomal irinotecan; Cap, capecitabine; CRTx, chemoradiation; MMC, mitomycin C.

For the complete overview of administered therapies subdivided by the two cohorts, we refer to Figure 1 and Table 2.

In first line systemic therapy, Gem/NabP ranks in both cohorts on top, however, relative frequencies differ substantially (26% in cohort A versus 69% in cohort B). Modified FOLFIRINOX (mFOLFIRINOX, no bolus 5-FU) was the second commonly used regimen in the two cohorts (19% vs. 15%) followed by Gem alone (14% versus 8%). In cohort A, further commonly used regimens were gemcitabine and oxaliplatin (Gem/Ox) (12%), oxaliplatin plus erlotinib (Gem/Ox/Erlotinib) (12%), chemoradiation (CRTx) (11%) and other therapies (6%, see Table 2), whereas, in cohort B further treatments were subsumed to other therapies, due to their low frequencies (8%, see Table 2).

The proportion of patients which started a second line therapy was 65% (*n* = 86) in cohort A and 62% (*n* = 104) in cohort B (see Figure 1). In descending order of relative frequencies, administered therapies in cohort A were Gem/NabP (21%), CRTx (19%), fluoropyrimidines and oxaliplatin (FP/Ox) (14%), gemcitabine/erlotinib (Gem/Erlotinib) (10%), Gem alone (7%), Gem/Ox (6%), mFOLFIRINOX (5%), capecitabine (Cap) (5%) and other therapies (14%). For cohort B these were nanoliposomal irinotecan and fluoropyrimidines (FP/Nal-IRI) (30%), CRTx (18%), FP/Ox (18%), Gem/NabP (13%), mFOLFIRINOX (5%), Gem/Ox/Erlotinib (5%), Cap (5%) and other therapies (7%).

Rates for administered third line therapies were 29% (*n* = 38) in cohort A and 37% (*n* = 63) in cohort B. In cohort A these therapies were Gem/NabP (26%), fluoropyrimidines and irinotecan (FP/Iri) (21%), FP/Ox (13%), Gem/Erlotinib (5%), mFOLFIRINOX (5%), Gem alone (5%), CRTx (5%), local to the site of the tumor directed therapies (5%) and other therapies (13%). In cohort B third line therapy consisted of FP/Ox (25%), FP/Nal-IRI (22%), Gem/NabP (16%), FP/Iri (10%), mFOLFIRINOX (6%), Gem/Ox (6%), Gem alone (5%) and other therapies (10%).

### 3.3. Clinical Outcome

**Figure 2:**
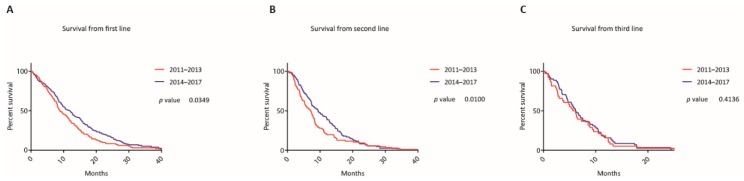
Survival according to treatment lines. Kaplan-Meier curves of patients from initiation of the first (**A**), second (**B**) and third (**C**) line treatment according to the time periods when the first systemic treatment was administered.

Kaplan-Meier curves for the two cohorts are depicted in Figure 2.

**Figure 3:**
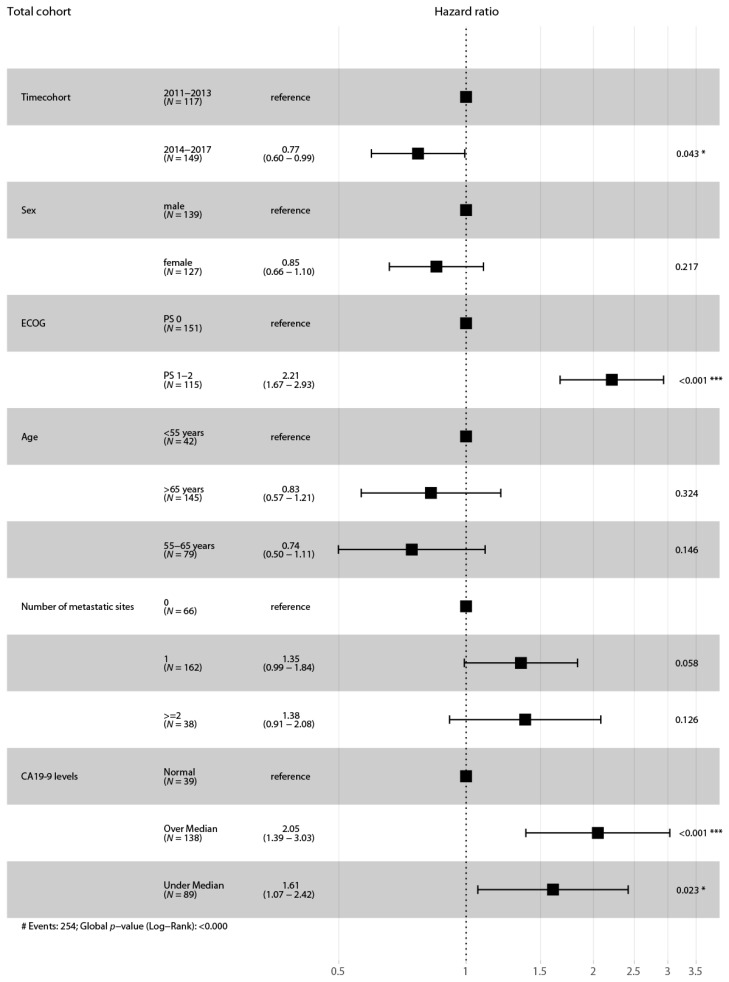
Forest plot for the adjusted survival analysis for all patients. Cox proportional hazard model to analyze the effect of multiple variables on the survival outcome of all patients. Abbreviations: ECOG, Eastern Cooperative Oncology Group performance scale. *N* = 266 (patients with missing values of variables were excluded from the analysis). Asterisks indicate *p*-value, * 0.01 to 0.05, ** 0.001 to 0.01, *** 0.0001 to 0.001. # indicates number.

In cohort A, 132 events occurred (100%) and in cohort B 157 (93%). The mOS from the beginning of first line treatment was 8.89 months in cohort A and statistically significantly longer in cohort B by 3.01 months (mOS 11.9 months; *p* = 0.035 (LRt), hazard ratio (HR) 0.77, confidence interval (CI) 0.61 to 0.98). The proportions of patients who were alive at 12, 18 and 24 months after initiation of first line therapy were 37.1% versus 49.1%, 16.7% versus 28.7% and 8.3% versus 17.3%. Multivariate analysis confirmed that the time when the first treatment line was initiated is a prognostic factor for survival (HR 0.77, CI 0.60-0.99, *p* = 0.043) (see Figure 3).

**Figure 4:**
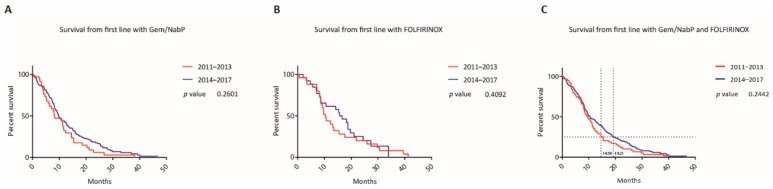
Survival between most popular first line regimens between the two time periods. Kaplan-Meier curves of patients who started a first line therapy with gemcitabine/nab-paclitaxel (**A**), mFOLFIRINOX (**B**) or with either one of these regimens (**C**) according to the year when the first systemic treatment was administered. Horizontal grid lines indicate the survival proportion of 25% and intersections with vertical grid lines indicate the survival time for the respective Kaplan-Meier curves at this point.

From beginning of second line therapy, mOS was 7.14 months versus 9.39 months (*p* = 0.010 (GBWt), HR 0.77, CI 0.57 to 1.04) and from the beginning of third line therapy, mOS was 5.89 months versus 6.15 months (*p* = 0.414 (LRt), HR 0.89, CI 0.59 to 1.34). Gem/NabP and mFOLFIRINOX were the most commonly administered first line therapies in both cohorts and accounted for 67% of all first line therapies from 2011 to 2017. When comparing these first line regimens between the two time cohorts, mOS did not differ significantly for first line Gem/NabP (8.23 months versus 9.82 months, *p* = 0.260 (LRt), HR 0.75, CI 0.49 to 1.14), mFOLFIRINOX (10.38 months versus 16.14 months, *p* = 0.409 (LRt), HR 0.84, CI 0.48 to 1.49) and Gem/NabP or mFOLFIRINOX (9.62 months versus 10.61 months, *p* = 0.244 (LRt), HR 0.79, CI 0.57 to 1.09) (see Figure 4).

**Figure 5:**
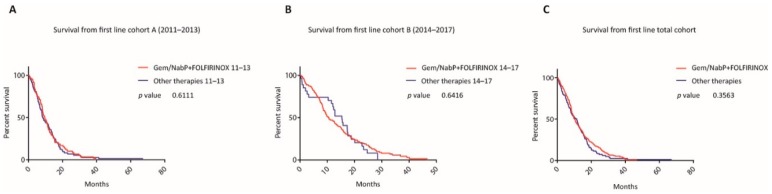
Survival with most popular first line regimens versus all other therapies within the two time periods. Kaplan-Meier curves of patients who started a first line therapy with either one of the two regimens gemcitabine/nab-paclitaxel and mFOLFIRINOX compared to all other therapies between 2011–2013 (**A**), 2014–2017 (**B**), as well as for the whole time period (**C**).

In neither time cohort nor in the whole cohort from 2011 to 2017, patients treated with these two first line regimes differed substantially in survival when compared to all other therapies (mOS in cohort A 9.62 versus 8.46 months, *p* = 0.611 (LRt), HR 0.96, CI 0.68 to 1.35; in cohort B 10.61 versus 15.37 months, *p* = 0.641 (LRt), HR 0.98, CI 0.64 to 1.51; in the total cohort 10.18 versus 11.01 months, *p* = 0.356 (LRt), HR 0.88, CI 0.69 to 1.13) (see Figure 5).

**Figure 6:**
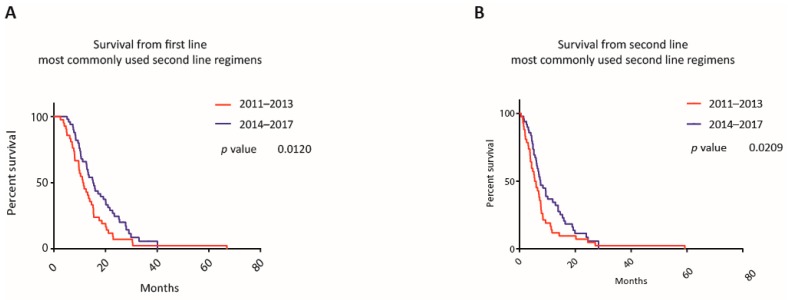
Survival according to first line and second line treatment for most popular regimens. Kaplan-Meier curves of patients who were treated with one of the most popular second line treatment regimes, calculated from the initiation of the first (**A**) and second (**B**) treatment line according to the year when the first systemic treatment was administered. The most popular treatment lines were gemcitabine/nab-paclitaxel, gemcitabine/(capecitabine)/erlotinib, as well as fluoropyrimidines/oxaliplatin for the cohort 2011–2013 and fluoropyrimidines/nanoliposomal irinotecan, as well as fluoropyrimidines/oxaliplatin for the cohort 2014–2017.

The ratio of patients who started a second line therapy was similar in both cohorts, however, the frequency of used regimens, unlike to first line regimens, was different. When excluding CRTx, which was applied in both cohorts at comparable rates (19% versus 18% in second line), the survival from first line and second line of patients treated with the most commonly used second line regimens (cohort A: 21% Gem/NabP, 14% Gem/(Cap)/Erlotinib, 14% FP/Ox; cohort B: 30% FP/Nal-IRI, 18% FP/Ox) differed significantly (mOS from first line 11.41 versus 15.21 months, *p* = 0.012 (LRt), HR 0.64, CI 0.41 to 0.99; mOS from second line 5.72 versus 7.47 months, *p* = 0.021 (GBWt), HR 0.68, CI 0.44 to 1.05) (see Figure 6).

**Figure 7:**
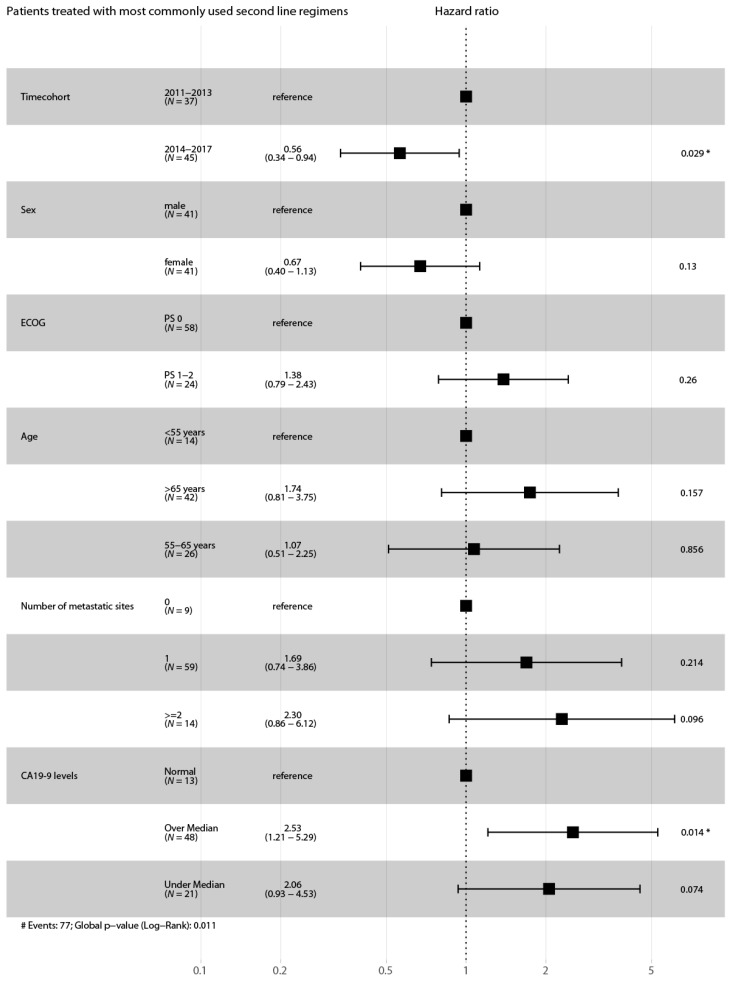
Forest plot for the adjusted survival analysis from patients treated with the most commonly used second line regimens of both time cohorts. Cox proportional hazard model to analyze the effect of multiple variables on the survival outcome of patients who have been treated with the most commonly used second line treatment regimens. Abbreviations: ECOG, Eastern Cooperative Oncology Group performance scale. *N* = 82 (patients with missing values of variables were excluded from the analysis). Asterisks indicate *p*-value, * 0.01 to 0.05. # indicates number.

Multivariate analysis (Cox proportional hazard model) with potentially influencing variables was performed for these two cohorts. The difference in survival between patients treated with the most commonly used second line regimens (2011–2013 vs. 2014–2017) remained statistically significant (HR 0.56, CI 0.34–0.94, *p* = 0.029) (see Figure 7).

**Figure 8:**
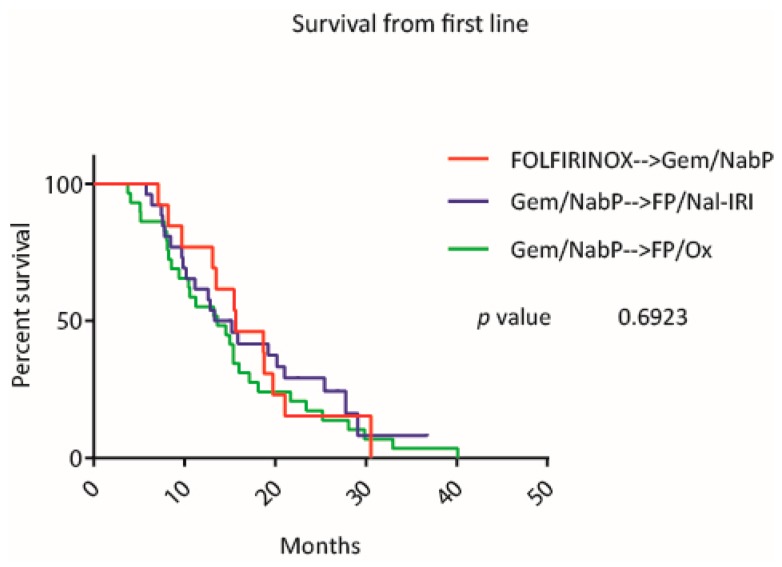
Survival with different first to second line treatment sequences. Kaplan-Meier curves of patients who were treated with one of the three treatment sequences from first to second line: mFOLFIRINOX followed by gemcitabine/nab-paclitaxel (red line), gem/nab-paclitaxel followed by fluoropyrimidines/nanoliposomal irinotecan (blue line) and gemcitabine/nab-paclitaxel followed by fluoropyrimidines/oxaliplatin (green line).

Among the total cohort, three different first to second line treatment sequences were analyzed. The survival curves for the sequences, mFOLFIRINOX followed by Gem/NabP (mOS 15.64 months), Gem/NabP followed by FP/Nal-IRI (mOS 14.27 months), Gem/NabP followed by FP/Ox (mOS 13.62 months), did not differ significantly from each other (*p* = 0.692) (see Figure 8).

## 4. Discussion

New therapeutic agents for metastatic PAC have recently expanded treatment options for this disease. Our aim was to assess the impact of these novel chemotherapy regimens on the treatment landscape and clinical outcome in a cohort of patients, which falls in the period of their introduction to the clinic. These treatment options include Gem/NabP, FOLFIRINOX, FP/Nal-IRI and FP/Ox. In concert with the timeline of clinical introduction of these regimens, we observed a major increase of Gem/NabP when comparing first line regimens in the period from 2011 to 2013 against 2014 to 2017 from 26% to 69% (*p* < 0.0001), while the rates for administration of mFOLFIRINOX as a front-line regimen differed not substantially (19% versus 15%, *p* = 0.4415). This increase of Gem/NabP in the first line setting was mainly accounted to a reduction of the other chemotherapy regimens Gem/Ox, Gem/Ox/Erlotinib and CRTx with less evidence as effective treatment options.

There have been two other studies from an American and a Japanese group assessing treatment patterns over recent years where modern first line chemotherapies were introduced and which showed a similar trend towards increased usage of Gem/NabP as a preferred first line regimen ranging from ~40% to 57% [16,17]. In contrast, another multi-institutional large study from Canada showed a tendency toward increased administration of FOLFIRINOX as first line treatment (60%) [18]. In countries, where S-1 is in clinical use for PAC, changes in frontline treatment patterns have also been observed. However, the use of Gem/NabP or FOLFIRINOX does not exceed 25% for each treatment option, according to two recently published studies from Japan and Taiwan [19,20]. Referring to our results regarding the second and third line setting, Gem/NabP was the most commonly used regimen in the earlier time period (21% and 26%), while, if considering the high rate of this regimen in the frontline setting of the later period, it was subsequently replaced by FP/Nal-IRI (30% and 22%). To sum up, when comparing the treatment landscape of the two time periods, we observed a clear increase of Gem/NabP, a regimen with high evidence as an effective front-line therapy with the later line treatment option FP/Nal-IRI.

Most importantly, the median survival of patients, who started their systemic treatment in the later time period, improved when compared to the earlier one (mOS 11.9 versus 8.89, *p* = 0.035) with survival rates at 12, 18 and 24 months after initiation of first line therapy of 37.1% versus 49.1%, 16.7% versus 28.7% and 8.3% versus 17.3%. This survival benefit was still evident when comparing the survival rates from the beginning of second line of the patients who started their first line therapy between 2011 and 2013 (mOS 7.14 versus 9.39 months, *p* = 0.010), but not from the beginning of third line. We analyzed if the increased ratio of first line treatment options with higher evidence as an effective therapy in the later time period could be responsible for the improvement in survival, but did not observe a difference when comparing Gem/NabP or mFOLFIRINOX, as well as both regimens to all other therapies among the two time cohorts and the total cohort (see Figure 5). There was also no significant survival difference between patients who started Gem/NabP or mFOLFIRINOX, as well as with either one of these two regimens among the two-time cohorts (see Figure 4). However, when comparing later time points of these survival curves, it was apparent that the survival curves separated at a time point where most patients already started a second line chemotherapy (compare intersection of indicated grid lines with survival curves from Figure 4C). In detail, at the time point of mOS of the respective time cohort, 90% and 84% of the patients who started a systemic therapy between 2011 to 2013 and 2014 to 2017 and also received a second line treatment already had begun this second or later treatment line. Next, we compared the survival of patients who received a second line treatment with the most commonly used regimens (cohort A: 21% Gem/NabP, 14% Gem/(Cap)/Erlotinib, 14% FP/Ox; cohort B: 30% FP/Nal-IRI, 18% FP/Ox) and observed that indeed there was a difference in survival time from first and second line treatment between the time cohorts (mOS from first line 11.41 versus 15.21 months, *p* = 0.012; mOS from second line 5.72 versus 7.47 months, *p* = 0.021). This survival benefit remained significant in the multivariate model when comparing patients who have been treated with the most commonly used second line regimens of the respective time cohorts (HR 0.56, CI 0.34–0.94, *p* = 0.029). These results suggest, that a change in treatment patterns, in particular that Gem/NabP moved from the second and third line to the first line setting in the later cohort and that FP/Nal-IRI was introduced as an effective second line regimen, was responsible for the observed survival benefit at our institution. Our hypothesis is strengthened by other reports. A recent study by Kordes M et al. reported that the sequence of gemcitabine-based therapy after 5-FU/oxaliplatin with or without irinotecan was associated with poorer outcomes than a 5-FU-based treatment after a gemcitabine-based doublet therapy [21]. Another study by Caparello and colleagues reports for the subgroup of patients who received Gem/NabP after the failure of first line FOLFIRINOX disappointing results in terms of both activity (RR: 7%; DCR: 23%) and survival (median PFS: 1.95 months; median OS: 5.4 months) [22]. Likewise, also other studies report only modest activity of second line Gem/NabP with median overall survival rates of 5.69 months and 5.75 months [23,24]. In descending order after Gem/NabP another frequently used second line therapy option in the earlier cohort was Gem/Erlotinib. In light of missing data concerning the effectiveness of Gem/Erlotinib as a second line treatment regimen and considering the only very modest improvement by the addition of erlotinib to gemcitabine reported in the literature, we believe that this regimen should not be considered as an effective second line treatment option [10,25]. In contrast to that, the most commonly used second line regimens in the earlier cohort are proven effective options as second line therapies, which has been shown particularly for FP/Nal-IRI [13]. This is also reflected by current treatment guidelines which mainly suggest FP/Nal-IRI after the failure of a gemcitabine-based first line chemotherapy.

There are limited data concerning the impact of new chemotherapy regimens on the outcome of patients with advanced or metastatic PAC in Caucasians, highlighting the main findings of our study. We are aware of another study conducted in a Taiwanese cohort, which also observed improved survival over a seven-year time period up to 2016 [20]. However, the authors associated this development to the increased use of S-1, which is not in clinical use in non-Asian countries, and thus, makes the results difficult to compare.

In line with the increased survival of patients treated with modern chemotherapy regimens, we also noted that the ratio of patients who received a third line therapy increased from 29% to 37% over time. These numbers also show a positive trend towards a prolonged treatment period when compared to other studies, which evaluated historical treatment patterns from 2005 to 2015 in Europe, as well as the US and reported rates between 3 to 22% for third line treatment [16,26,27].

This opens up the discussion for the optimal treatment sequences. The mOS seen across the analyzed first to second line treatment sequences was encouraging, ranging from 13.62 to 15.64 months. Given that FOLFIRINOX is associated with a higher toxicity rate, it is noteworthy that the survival of patients who also received a second line therapy was comparable and did not differ significantly from patients, who had not been treated with mFOLFIRINOX in the first line. There are limiting data concerning the effectiveness of different treatment sequences, but a recently published study, which evaluated the use of Gem/NabP followed by mFOLFIRINOX or vice versa in 48 patients did not report any difference in survival between the two groups (mOS 13.7 and 13.8 months, *p* = 0.9) [28]. Supporting these and our findings, also a study by Glassman and colleagues reported promising survival rates for the sequence Gem/NabP followed by FP/Nal-IRI (mOS 23.0 months). They did not find a significant difference to the sequence FOLFIRINOX followed by Gem/NabP and FP/Nal-IRI (mOS 25.5 months) [29]. Furthermore, in the previously mentioned study by Kordes et al. patients who received FOLFIRINOX had a shorter median OS (9.9 months, 95% CI; 8.1–11.7) than previously reported [21]. In that respect and in light of no head-to-head prospective comparison trials, there is still no evidence that FOLFIRINOX in the palliative setting is associated with increased survival compared to Gem/NabP. This triplet-therapy, however, is regarded more toxic when compared to doublet-regimens, which should be considered in particular if palliation is the primary goal of the treatment.

Our study has several limitations, including being conducted as a single-institution analysis, the relatively small sample size of the different sequence subgroups, the inclusion of patients only when they were presented to the multidisciplinary tumor board, its retrospective character, as well as missing data for adverse events and toxicity. Furthermore, we cannot rule out a possible bias resulting from potential earlier switching to second line chemotherapy in the earlier time cohort, due to closer monitoring of disease activity. However, we have no reason to believe that this has occurred because standard diagnostic and therapeutic work-up, including intervals between reassessment of treatment responses have not changed during the observational period. Moreover, provision of better supportive therapy, which would allow more patients to complete systemic treatment for advanced-stage PAC could have impacted the results of our study. In this respect, there might also be additional influencing factors like better handling of dose adjustments when encountering toxicities. These potential influencing factors are, however, not very likely to explain the improved survival between our cohorts, as individual components of the different chemotherapies like platinum salts, fluoropyrimidines, irinotecan, gemcitabine, as well as erlotinib and nab-paclitaxel are classic antineoplastic drugs which were already in use long before the observation period of this study started. Thus, it is not very likely that our clinical management concerning toxicities and dose adjustments has changed during 2011 and 2017.

Emerging promising predictive biomarkers like mismatch-repair deficiency (or MSI-high) for checkpoint blockade or BRCA mutations for PARP inhibitors and platinum salts are currently entering the clinic, but have not been used for any patient in the current study [30,31]. With the year 2018, our cancer center started to implement testing for mismatch-repair deficiency and BRCA mutations of tumors from PAC patients. Although, only a small subset of patients is affected by these conditions, it is an important step towards personalized treatment of PAC patients, which will hopefully improve the prognosis and add new effective treatment options.

## 5. Conclusions

This study describes the changes in the treatment landscape in the clinical management of patients with unresectable PAC and furthermore underscores the effectiveness of the new chemotherapy regimens, which hopefully invigorates the discussion about optimal treatment sequencing, as well as the choice of the optimal frontline regimen. It is encouraging that survival rates have increased at our institution. This positive trend is most likely explained by the introduction of novel treatment options. Furthermore, the high rate of patients that started a third line therapy (37%) is stimulating. Additional analyses of sequential treatment in a sufficiently large study population are warranted.

## Figures and Tables

**Table 1:** Patient Characteristics of patients and tumors of the two main study cohorts.

	Cohort A 2011–2013 (*n* = 132)	Cohort B 2014–2017 (*n* = 169)	*p*-Value
Median age at diagnosis of advanced disease (median, range)	66.3 (57.3–72.2)	66.0 (58.3–73.3)	ns
Gender (%)			
Female	53 (41)	90 (53)	*
Male	79 (59)	79 (47)	
Disease stage (%)			
Locally advanced (unresectable)	34 (26)	43 (25)	ns
Metastatic	98 (74)	126 (75)	
Prior surgical resection (%)	23 (17)	32 (19)	ns
Median CA 19-9 levels in kU/l (range)	505.35 (68.05–2261.25)	686.40 (88.25–3771.00)	ns
CA 19-9 levels (%)			
Within normal range	20 (15)	19 (11)	ns
Above normal range	101 (77)	132 (78)	
*n*/a	11 (8)	18 (11)	ns
Site of metastatic disease (%)			
Liver	73 (55)	84 (50)	ns
Lung	15 (11)	21 (12)	ns
Peritoneum	10 (8)	30 (18)	*
Other	11 (8)	7 (4)	ns
Number of metastatic sites (%)			
0	34 (26)	43 (25)	ns
1	80 (60)	102 (60)	ns
2	14 (11)	18 (11)	ns
≥3	4 (3)	6 (4)	ns
ECOG Performance Status (%)			
0	67 (51)	100 (59)	ns
1	52 (39)	61 (36)	ns
2	8 (6)	5 (3)	ns
n/a	5 (4)	3 (2)	ns

Abbreviations: CA 19-9, Carbohydrate antigen 19-9; ECOG, Eastern Cooperative Oncology Group; ns, not significant (*p* > 0.05).

**Table 2:** Administered chemotherapy regimens.

	First Line		*p*	Second Line		*p*	Third Line		*p*
Chemotherapy	2011–2013	2014–2017		2011–2013	2014–2017		2011–2013	2014–2017	
Gem/NabP	34 (26%)	116 (69%)	****	18 (21%)	13 (13%)	ns	10 (26%)	10 (17%)	ns
mFOLFIRINOX	25 (19%)	26 (15%)	ns	4 (5%)	5 (5%)	ns	2 (5%)	4 (7%)	ns
Gem/Ox	16 (12%)	5 (3%)	**	5 (6%)	2 (2%)	ns	0 (0%)	4 (7%)	ns
Gem/Ox/Erlotinib	16 (12%)	0 (0%)	****	3 (3%)	5 (5%)	ns	1 (3%)	0 (0%)	ns
Gem alone	18 (14%)	14 (8%)	ns	6 (7%)	2 (2%)	ns	2 (5%)	3 (5%)	ns
Gem/Erlotinib	4 (3%)	0 (0%)	*	9 (10%)	1 (1%)	**	2 (5%)	2 (3%)	ns
FP/Ox	1 (1%)	2 (1%)	ns	12 (14%)	19 (18%)	ns	5 (13%)	16 (28%)	ns
FP/Iri	1 (1%)	0 (0%)	ns	1 (1%)	2 (2%)	ns	8 (21%)	6 (10%)	ns
FP/Nal-IRI	0 (0%)	1 (1%)	ns	0 (0%)	31 (30%)	****	0 (0%)	14 (24%)	****
Cap	0 (0%)	1 (1%)	ns	4 (5%)	5 (5%)	ns	1 (3%)	2 (3%)	ns
Gem/Cap	0 (0%)	1 (1%)	ns	2 (2%)	0 (0%)	ns	0 (0%)	2 (3%)	ns
Ox	1 (1%)	0 (0%)	ns	0 (0%)	0 (0%)	ns	1 (3%)	0 (0%)	ns
CRTx	15 (11%)	3 (2%)	***	16 (19%)	19 (19%)	ns	2 (5%)	0 (0%)	ns
MMC	0 (0%)	0 (0%)	ns	1 (1%)	0 (0%)	ns	1 (3%)	0 (0%)	ns
Local therapy	1 (1%)	0 (0%)	ns	2 (2%)	0 (0%)	ns	2 (5%)	0 (0%)	ns
Gem/Cap/Erlotinib	0 (0%)	0 (0%)	ns	3 (3%)	0 (0%)	ns	1 (3%)	0 (0%)	ns
No therapy	0 (0%)	0 (0%)	ns	46 (35%)	65 (38%)	ns	94 (71%)	106 (63%)	ns

Abbreviations: Gem/NabP, gemcitabine/nab-paclitaxael; mFOLFIRINOX, modified 5-FU, leucovorin, irinotecan, and oxaliplatin; Gem/Ox, gemcitabine/oxaliplatin; FP/Ox, fluoropyrimidines/oxaliplatin, FP/Iri, fluoropyrimidines/irinotecan; FP/Nal-IRI, fluoropyrimidines/nanoliposomal irinotecan; Cap, capecitabine; CRTx, chemoradiation; MMC, mitomycin C, *p*, *p*-value; ns, not significant (*p* > 0.05). Asterisks indicate *p*-value, * 0.01 to 0.05, ** 0.001 to 0.01, *** 0.0001 to 0.001, **** < 0.0001.
